# Preliminary Study on Hepatoprotective Effect and Mechanism of (-)-Epigallocatechin-3-gallate against Acetaminophen-induced Liver Injury in Rats

**DOI:** 10.22037/ijpr.2020.112727.13918

**Published:** 2021

**Authors:** Yongxu Lin, Juan Huang, Tingfang Gao, Yuanzi Wu, Da Huang, Fen Yan, Zuquan Weng

**Affiliations:** a *Department of Basic Education, Fujian Normal University, Fuzhou 350116, China. *; b *College of Biological Science and Engineering, Fuzhou University, Fuzhou 350116, China.*; 1 *These authors contributed equally to this work.*

**Keywords:** EGCG, APAP, Liver Injury, Mitochondria, ROS

## Abstract

Antipyretic acetaminophen (APAP) is a commonly used drug that generally associates with liver injury. (-)-Epigallocatechin-3-gallate (EGCG), an active polyphenol extracted from green tea, is extensively reported to have the potential to impact a variety of human diseases. However, few studies were reported regarding the protective effect of EGCG on APAP-induced liver injury and the mechanism is still unclear. In this study, *in-vitro* and *in-vivo* experiments were carried out to verify the hepatoprotective effect of EGCG against APAP-induced liver injury and explore the potential mechanism. Results indicated that EGCG effectively relieved the liver injury caused by APAP, as well as APAP-induced mitochondrial dysfunction. The protective role of EGCG was not only attributed to its antioxidant capacity; but also might be related to the protective effect on hepatic mitochondrial impairment; based on that, EGCG could improve the membrane potential and activities of the respiratory chain complexes in liver mitochondria. Our study casts a new light on the mechanism of EGCG’s hepatoprotective effect and suggests that EGCG has considerable potential in developing tonics for relieving APAP-induced liver injury.

## Introduction

Adverse drug reaction (ADR) is a potential complication of any pharmacotherapy, and drug-induced liver injury is one of the most frequent and serious ADRs since liver is the major target organ for drug metabolism (1-5). According to information statistics, more than 1100 currently available drugs can lead to liver injury (6-8). Hence, drug-induced liver injury has a considerable impact on human health and has become a non-negligible public health problem.

Antipyretic acetaminophen (APAP) is a commonly used antipyretic and analgesic drug. However, overdosage of APAP will induce acute liver failure due to its highly reactive intermediate *N*-acetyl-*p*-benzoquinone imine (NAPQI), mainly catalyzed by cytochrome P450 (CYP) 2E1 and 3A4 (9-11). NAPQI can react with the sulphydryl groups of glutathione (GSH), resulting in cellular GSH depletion and the formation of reactive oxygen species (ROS) in the liver (12, 13). Excessive ROS are positively correlated with mitochondrial impairment, which has long been considered as an important mechanism for drug or chemical-induced liver injury (14). In addition, NAPQI can covalently conjugate to proteins and produce NAPQI-protein adducts after GSH depletion, which will also cause serious hepatotoxicity since mitochondrial proteins can be one of the chief targets attacked by NAPQI (15, 16). Therefore, APAP-induced liver injury may be highly related to mitochondrial impairment caused by NAPQI.

Several studies have demonstrated that some natural products, such as (-)-Epigallocatechin-3-gallate (EGCG), may relieve APAP-induced liver injury, but the relative mechanism is not fully clear (17-20). EGCG is the major tea flavonoid with multifarious alleged pharmacological effects and is commonly used as an essential component of many functional foods, such as antioxidants and weight loss aides (21-26). Recently, the protective effect of EGCG on drug-induced liver injury has aroused great interest of researchers. Yang and co-workers discovered that EGCG profoundly influenced hepatic CYPs, probably resulting in changed pharmacokinetics and toxicity of drugs (27). Bradshaw and co-workers reported that EGCG treatment restored Alzheimer’s amyloid-induced decrease of mitochondrial respiratory rates, mitochondrial membrane potential, ROS production, and adenosine triphosphate (ATP) levels by 50~85% (28).

Despite many research projects focused on the EGCG’s protective effect on hepatic damage, only a few studies were related to its function to APAP-induced acute liver injury. Recently, Yin and co-workers (18) reported that EGCG significantly reduced APAP-induced liver injury in rats, possibly due to EGCG lowering the activities of CYP enzymes, such as CYP3A4 and CYP2E1 (29). However, there is a lack of information about the protective effect and potential mechanism of EGCG against APAP-induced hepatic mitochondrial impairment. 

Herein, the effect of EGCG on APAP-induced liver injury and hepatic mitochondrial impairment was investigated by comparing hepatic biological parameters and mitochondrial functions when rats and isolated liver mitochondria with the treatment of APAP or both APAP and EGCG. This study may provide more information about the mechanism of EGCG’s protective effect on APAP-induced liver injury.

## Experimental

*Materials*

APAP, nicotinamide adenine dinucleotide (NADH), antimycin A, ubiquinone 1, sodium succinate, rotenone, 2,6-dichloroindophenol sodium, phospho(enol)pyruvic acid monopotassium salt, lactate dehydrogenase (LDH), ATP and oligomycin A were obtained from Sigma-Aldrich (St. Louis, MO, USA). All other chemicals with analytical grade were purchased from Sinopharm Chemical Reagent Co., Ltd (Shanghai, China). EGCG (>98% pure) was provided by Shanghai Ronghe Pharmacy Co., Ltd. (China).

*Animals*

Male Sprague-Dawley (SD) rats (230-280 g, Shanghai Lingchang Biotechnology Co., Ltd. China) with one group of six were used for this study, and the experimental design consisted of eight groups. In four groups of them, the 100 mg/kg EGCG as daily dose was administered to rats by gavage for five consecutive days, and the control group was fed with normal saline. Subsequently, three groups with the treatment of EGCG and another three groups without the treatment of EGCG were administered with 250, 500 and 1000 mg/kg APAP, respectively, by intraperitoneal injection. After 8 h, all rats were killed by CO_2_. Blood samples from the postcaval vein were collected into coded tubes containing anticoagulant EDTA and then were used for the extraction of plasma by centrifugation. The liver tissues were isolated and stored at -80 C. Partly liver samples were fixed in 10% neutral buffered formalin and then processed histological assessment with H-E staining. All experimental protocols employed herein were approved by the Committee on the Care of Laboratory Animal Resources, College of Biological Science and Engineering, Fuzhou University.

*Methods*

*Measurement of alanine aminotransferase (ALT) and LDH activity*

ALT and LDH in plasma were measured by ALT assay kit (Beckman Co., Ltd, German) and LDH assay kit (Shanghai Shenneng Biotechnology Co., Ltd, China), respectively.

*Measurement of MDA, SOD and GSH activity*

The levels of oxidative stress -malondialdehyde (MDA) and biomarkers for antioxidant- superoxide dismutase (SOD) and GSH were measured using a commercially available kit (Nanjing Jiancheng Bioengineering Institute, Nanjing, China). In brief, partly liver of rats was homogenized in ice-PBS, then the samples were centrifuged at 3000 g for 10 min at 4 C. The supernatants were used for the measurement of MDA, SOD, and GSH activities according to the manufacturers’ instructions.

*Isolation of rat hepatic mitochondria*

Rat liver mitochondria were prepared as the description of the previous report with minor modification (30). After being washed by isolation buffer (70 mM sucrose, 190 mM mannitol, 20 mM HEPES, 0.2 mM EDTA, pH 7.5), the liver was homogenized using a 100 ml Dounce type homogenizer. Then the concentrations of mitochondrial proteins were determined by the Bradford kit of Bio-Rad (Hercules, CA, USA).

*Treatment of isolated liver mitochondria from untreated rats*

After liver mitochondria from untreated rats were isolated according to the methods mentioned above, mitochondrial preparations (1 mg/mL) were incubated with 0, 3.75, 7.5, 15, 30, 45, 60, and 100 µM EGCG at room temperature for 30 min. On the other hand, APAP was dissolved in DMSO and added to final concentrations of 0, 20, 40, 60, 80, 100 mM in mitochondria preparations. The final DMSO concentration was 5‰. Before it was treated with 60 mM APAP for 5min, the mitochondria were incubated with 30, 60 and 100 μM EGCG for 30 min prior to downstream experiments.

*Detection of mitochondrial membrane potential (MMP)*

The analysis of MMP in liver tissues was determined by the Sigma-Aldrich (St. Louis, MO, USA) JC-10 Assay Kit according to the kit’s instructional manual, and the excitation wavelength was 488 nm, and the emission wavelengths were 530 nm and 590 nm. The fluorescence intensity ratio between 590 nm and 530 nm was calculated to reflect changes in MMP. At the same time, the changes of the membrane potential of mitochondria dyed by JC-10 were observed by a confocal fluorescence microscope (CTR6500, Leica) as described previously (31).

*Measurement of respiratory chain complexes (RCCs) activities*

Mitochondria treated with EGCG and/or APAP were centrifuged and re-suspended in 25 mM potassium phosphate (pH 7.2) and subjected to three cycles of freezing-and-thawing. The liver mitochondria treated with EGCG and/or APAP *in-vitro* and administered with EGCG and APAP *in-vivo* were used for the measurement of RCC I–V activities. The method is carried out according to a reported protocol (32), except that a smaller reaction volume of 0.2 mL was used.

*Statistical analysis*

SPSS statistical package (SPSS, Chicago, USA) was used to perform statistical analysis. Effect of APAP exposure on LDH, ALT, MDA, SOD, GSH, complex I and complex III in rats was performed using one-way analysis of variance (ANOVA). Student’s *t*-test was applied for comparisons between averages of two samples. For the histopathological data, the significance of a decreased incidence of centrilobular hypertrophy and granuloma of livers in the APAP-treated groups pre-incubated with EGCG, when compared to the corresponding APAP-treated groups without incubation of EGCG, was evaluated by Fisher’s exact test. The minimal of level of significance chosen was *p* < 0.05.

## Results

*In vitro evaluation of the protective effect of EGCG on APAP-induced mitochondrial impairment*

**Figure 1:**
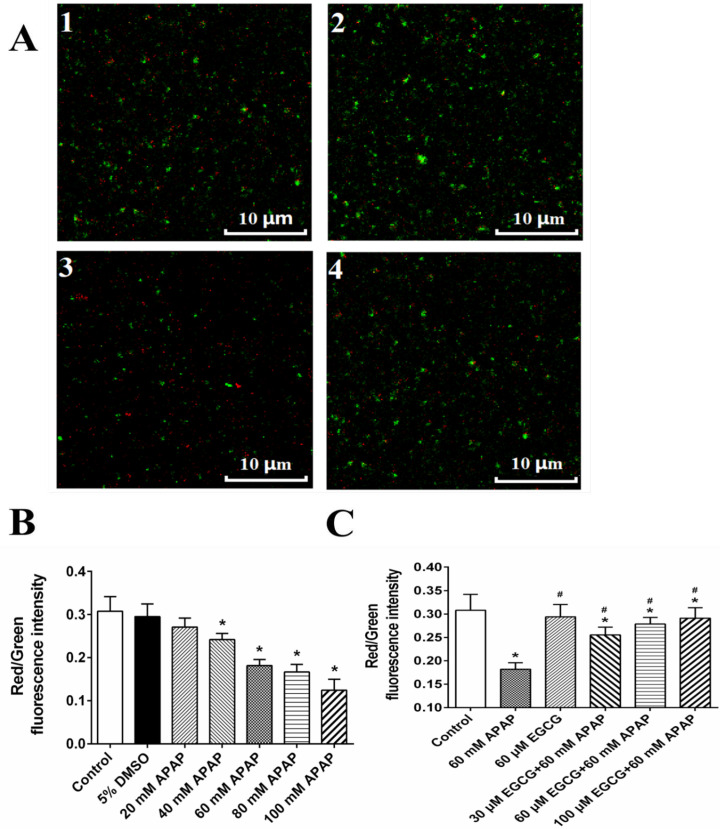
Effect of APAP on MMP and protective role of EGCG *in-vitro*. A: MMP observed by confocal fluorescence microscope; and 1: control; 2: 60 mM APAP; 3: 60 μM EGCG; 4: 60 μM EGCG + 60 mM APAP. The quantitative data about the ratio of red and green fluorescence were presented in panels B and C. The data are mean ± SD, n = 3. In B, ^*^p < 0.05, compared to 5% DMSO; in C, ^*^p < 0.05, compared to control, and ^#^*p* < 0.05, compared to 60 mM APAP

MMP of rat livers was evaluated by JC-10 assay (Figure S1A) and confocal microscopy (Figure 1B), and results of two methods showed that APAP could significantly result in the decrease of red/green ratio representing the depolarization of MMP. APAP-induced depolarization of MMP might be recovered by EGCG (Figure S1B and Figure 1C), when mitochondria were pre-incubated with EGCG before the 60 mM APAP treatment.

**Figure 2:**
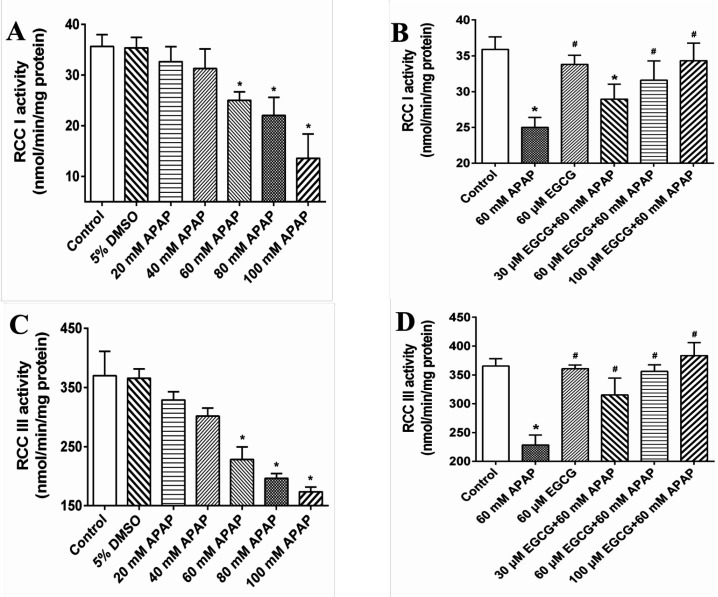
Effect of APAP on RCC I and III and protective role of EGCG *in-vitro*

In Figures 2A and 2C, we further observed that APAP exposure had significant effects on decreasing RCC I and III activities, but not in RCC II, IV and V (Data not shown). When pre-treated with EGCG prior to APAP, the RCC I and III activities loss caused by APAP were significantly ameliorated (Figures 2B and 2D).

*In-vivo evaluation of the protective effect of EGCG on APAP-induced liver injury in rat*

**Figure 3:**
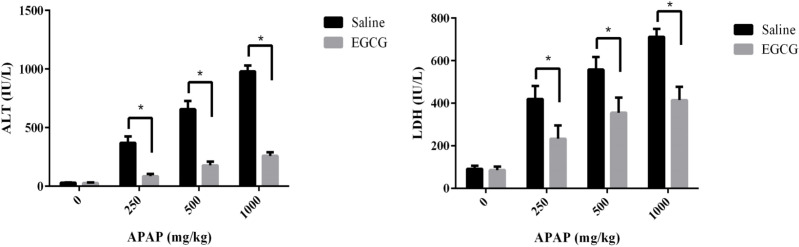
(A) ALT and (B) LDH levels in the plasma of SD rats with/without EGCG pre-treatment after being injected APAP for 8 h. Values represent the mean ± SD. ^*^p < 0.05, based on student's* t*-test

After being treated with or without EGCG (100 mg/kg) by gavage, all rats were exposed to a different dosage of APAP. Then the plasma ALT and LDH levels of SD rats were measured to evaluate the effect of EGCG on the liver injury caused by APAP. As summarized in Figure 3, no matter whether the rats were pre-treated with or without EGCG, their plasma ALT and LDH levels increased significantly after being injected APAP for 8 h, compared to the control group without APAP treatment. Furthermore, with an increase in the APAP dosage, the plasma ALT and LDH levels of both groups went up. However, the plasma ALT and LDH levels in the rats fed with EGCG were much lower than rats without EGCG treatment at the same APAP dosage, suggesting that the EGCG remarkably restored the liver injury caused by APAP. 

**Figure 4:**
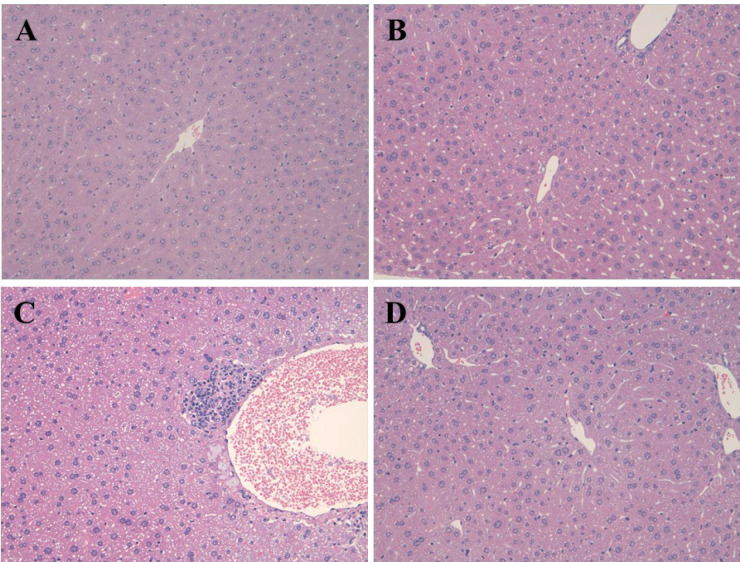
Photomicrophs (original magnification × 200) of H&E-stained livers in rats

Figure 4 demonstrated the pathological changes in the hepatic tissue of rats in the APAP, EGCG and EGCG + APAP groups. The statistical analysis of the histopathological findings in rat livers was summarized in Table 1. In accordance with the results of ALT and LDH, APAP significantly caused centrilobular hypertrophy and granuloma. The APAP-induced histopathological findings were significantly reduced by pre-treatment of EGCG.

**Figure 5:**
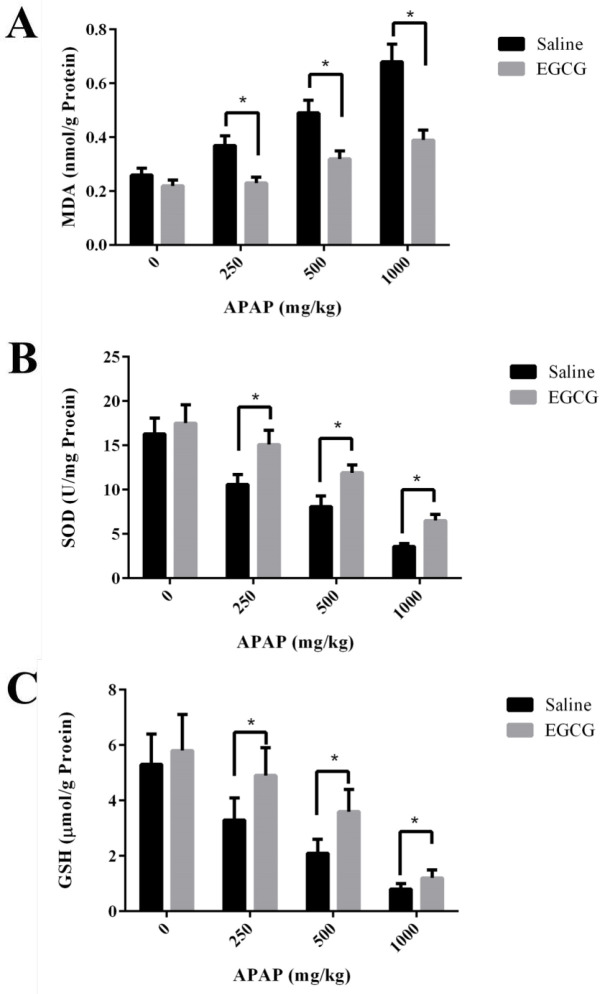
(A) MDA, (B) SOD and (C) GSH levels in the liver of SD rats with/without EGCG pre-treatment after being injected APAP for 8 h. Values represent the mean ± SD. *p < 0.05, based on student's *t*-test

Biomarkers for oxidative stress - MDA, and biomarkers for antioxidant - SOD and GSH in the liver of SD rats were measured. As shown in Figure 5A, MDA concentrations in the liver of SD rats without EGCG treatment increased remarkably after APAP injection. However, under the administration of the same APAP dosage, the groups with pre-treatment of EGCG have significantly lower concentrations of MDA than those of the groups without EGCG treatment. After APAP injection, evident reduction of SOD activities and GSH concentrations were observed in both groups with and without EGCG treatment, and the reduction was in a dosage-dependent manner (Figure 5B-C). However, the rats with EGCG treatment showed much higher SOD and GSH concentrations than those without EGCG treatment, indicating the administration of EGCG, significantly relieved the levels of oxidative stress. On the other side, there was no significant difference in GSH content among the two groups at the high dose of APAP (1000 mg/kg), which suggested that 100 mg/kg EGCG has less effect on 1000 mg/kg APAP-induced GSH depletion.

**Figure 6:**
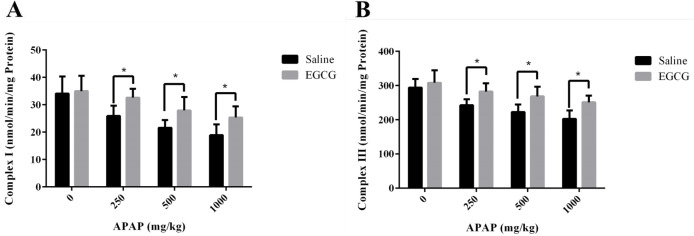
Activities of RCC (A) I and (B) III in liver mitochondria of SD rats with/without EGCG treatment after being injected APAP for 8 h. Values represent the mean ± SD. *p < 0.05, based on student's *t*-test

The activities of RCCs in liver mitochondria of rats treated with or without EGCG were measured to investigate the possible hepatic mitochondrial impairment caused by APAP. As demonstrated in Figure 6, both RCC I and III activities reduced with the increase of dosage of APAP, indicating hepatic mitochondrial injury caused by APAP. However, higher RCC I and III activities were observed in liver mitochondria of rats with EGCG treatment, manifesting that EGCG administration could recover the lower activities of mitochondrial RCCs induced by APAP.

The liver mitochondria of rats were administrated EGCG with different concentrations. Then the activities of all RCCs in liver mitochondria were determined and results were shown in Figure S3. In the range of 0 to 100 μM EGCG, data revealed hardly any significant influence on activities of all RCCs. These results turned out that only treated with EGCG did not affect the activities of RCCs in liver mitochondria remarkably.

## Discussion

Pathological changes in the liver and increases in serum ALT and LDH levels are the main characteristics of liver damage. The results of our study showed that APAP could significantly increase the damage degrees of centrilobular hypertrophy and granuloma in rat livers, and the levels of serum ALT and LDH in a dose-dependent manner, while pre-treatment of EGCG significantly decreased the elevation, demonstrating that EGCG is effective in alleviating hepatic injury caused by APAP. 

In addition to lower ALT and LDH levels, we also observed that the higher level of MDA in the liver of rats caused by the injection of APAP was reduced by pre-treating with EGCG. Moreover, EGCG could also reduce the depletion of GSH and SOD caused by APAP. It has been reported that APAP toxicity mediated by the reactive metabolite NAPQI, which was catalyzed by CYPs, mainly are CYP2E1 and 3A4 (33, 34). The previous study proved that EGCG could covalently bind to cysteinyl thiol groups in protein (35), and the following researches indicated that EGCG could reduce the formation of NAPQI, due to that the ability of EGCG to covalently bind to various CYPs and inhibit the activities of CYP2E1 and 3A4 (18, 29 and 36).

It should be noted that a small amount of NAPQI does not cause liver toxicity because it will be detoxified by combination with GSH, which is the original detoxifying substance in the liver. However, NAPQI will exhaust GSH by the reaction of GSH with excessive NAPQI after an APAP overdose (37), further leading to a lot of ROS accumulation in the body and eventually resulting in a higher level of MDA and a lower level of SOD (38). According to our results, EGCG administration followed by APAP markedly reduced MDA production and increased the levels of GSH and SOD, which exhibited that EGCG had a good antioxidant capacity, in accordance with the previous report (24). 

Many studies provided convincing evidence that mitochondrial ROS played a critical role in APAP-induced liver injury (14, 39 and 40). Besides the metabolism of APAP and accumulation of its toxic metabolite in the liver, the inhibitory effect of APAP and/or its metabolite on RCCs activities in liver mitochondria was another cause of liver injury (41-43). After GSH depletion, NAPQI would bind to the thiol side chains of cysteine residues in mitochondrial proteins, which might result in the inactivation of mitochondrial functions (44-48). And mitochondrial dysfunctions caused the generation of a large amount of ROS (49). Excessive ROS, in turn, would impair mitochondria, thus forming a vicious circle and finally led to hepatic impairment. Previously, Salminen and co-workers reported that green tea extract with 48.4% EGCG essentially eliminated the production of NAPQI covalently binding to proteins (50). The findings suggested that EGCG could prevent NAPQI from binding to mitochondrial proteins by its direct reaction with NAPQI, similar to the reaction of NAPQI with GSH. In our results, *in-vitro* study illustrated that EGCG pre-incubation could prevent APAP-induced mitochondrial damage, including the depolarization of MMP and decrease of RCC I and III, and the later results were further confirmed by the data obtained from *in vivo* study. These findings taken together showed that EGCG plays a protective role on APAP-induced liver injury by maintaining mitochondrial integrity. Therefore, we deduced that EGCG either scavenged the excessive ROS or prevented the formation of NAPQI and NAPQI-protein adducts, thereby protecting the integrity of mitochondrial function. Further basic studies are needed to verify this viewpoint. 

Miteshkumar and co-workers ever reported that *N*-acetylcysteine, hypotaurine and taurine could equally attenuate the hepatic injury (17). However, they are all drugs that may be associated with side effects, and in comparison to them, natural product EGCG is much safer. Our experiment results also showed that EGCG did not significantly affect the activities of RCCs, even at a very high EGCG dosage of 100 μM. In fact, the average maximum plasma concentration (C_max)_ of EGCG was 0.3-7.4 µM, some individuals had as high as 13.2 µM EGCG in the blood (51, 52), and this range concentrations of EGCG could not affect the RCCs activities. Therefore, EGCG is a promising candidate as tonics for relieving liver injury induced by APAP.

(A) control; (B) EGCG-only; (C) APAP-only; (D) EGCG+APAP.

**Table 1:** Incidence of pathological changes in liver tissues of SD rats with/without EGCG pre-treatment after being injected APAP for 8 h

**Histopathology**	**EGCG (mg/kg)**	**No. of Rat**	**Degrees of pathological changes**				
			**APAP (mg/kg)**				**Total (250+500+1000)**
			**0**	**250**	**500**	**1000**	
			**- ± + ++**	**- ± + ++**	**- ± + ++**	**- ± + ++**	**- ± + ++**
Centrilobular hypertrophy	0	6	6 0 0 0	3 2 1 0	0 2 3 1*	0 1 2 3*	3 5 6 4*
	100	6	6 0 0 0	4 2 0 0	3 2 1 0*	1 3 2 0*	8 7 3 0^#^
							
Ganuloma	0	6	6 0 0 0	4 1 1 0	1 1 3 1*	0 1 3 2*	5 3 7 3*
	100	6	6 0 0 0	5 1 0 0	4 1 1 0	2 3 1 0	11 5 1 0^#^

-: none; ±: extremely slight; +: slight; ++: moderate. ^*^p < 0.05, based on the Fisher's exact test, and significantly different from the corresponding group exposed to 0 mg/kg APAP; ^#^*p* < 0.05, based on the Fisher's exact test, and significantly different from the corresponding group exposed to 0 mg/kg EGCG.

## Conclusion

In conclusion, our study demonstrated that EGCG could effectively restore the APAP-induced liver injury by its antioxidant capacity and recovering the lower activities of RCCs in liver mitochondria caused by APAP. It is the first time to propose that the protective effect of EGCG on APAP-induced liver injury is due to EGCG can alleviate the hepatic mitochondrial impairment, probably via eliminating ROS and reducing the reactive intermediate (NAPQI) of APAP and NAPQI-protein adducts. We believe this study casts a new light on the mechanism of EGCG’s hepato-protective effect and suggests that EGCG has considerable potential in developing tonics for treating APAP-induced liver injury.
