# Supplementary material for: Preliminary Study on Hepatoprotective Effect and Mechanism of (-)-Epigallocatechin-3-gallate against Acetaminophen-induced Liver Injury in Rats
Source: Iran J Pharm Res. 2021 Summer;20(3):46–56. doi: 10.22037/ijpr.2020.112727.13918 (PMC8653645; doi:10.22037/ijpr.2020.112727.13918)
Supplement: Supplementary file 1 [file ijpr-20-46-s001.pdf]

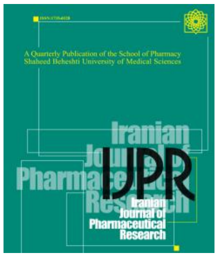

Supplementary Materials for

Preliminary Study on Hepatoprotective Effect and Mechanism of (-)-Epigallocatechin-3-gallate against Acetaminophen-induced Liver Injury in Rats

Yongxu Lin[1], Juan Huang[1], Tingfang Gao, Yuanzi Wu, Da Huang, Fen Yan* and Zuquan Weng*

*To whom correspondence should be addressed. E-mail: yanfen@fzu.edu.cn; wengzq@fzu.edu.cn

[1]These authors contributed equally to this work.

Volume 20, Issue 3 (Summer 2021)

This PDF file includes:
Figure S1-S3

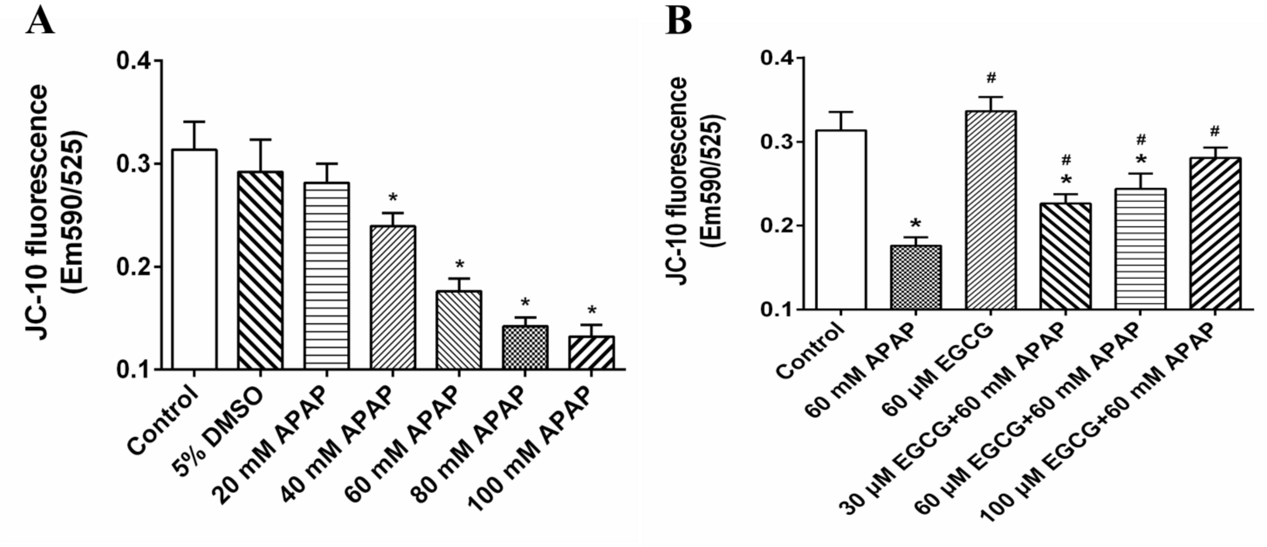

Figure S1. Evaluate the effects of APAP and EGCG on MMP in rat livers using JC-10 assay. The quantitative data about the ratio of Em590/525 were presented in panel A and B. The data are mean ± SD, n = 3. In A, *$p < 0.05$, compared to 5% DMSO; in B, *$p < 0.05$, compared to control, and $^{\#}p < 0.05$, compared to 60 mM APAP.

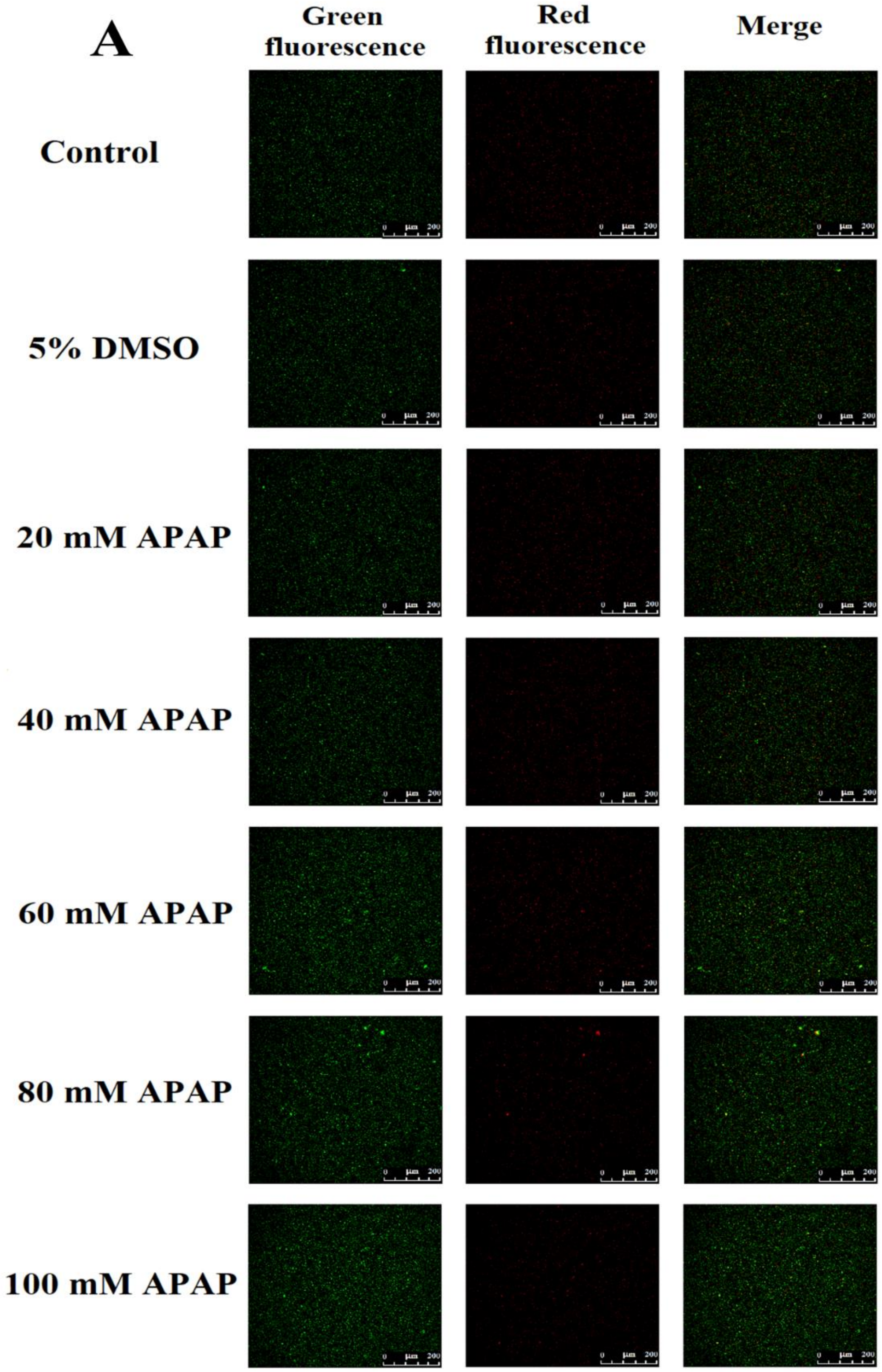
A
Green fluorescence
Red fluorescence
Merge
Control
5% DMSO
20 mM APAP
40 mM APAP
60 mM APAP
80 mM APAP
100 mM APAP

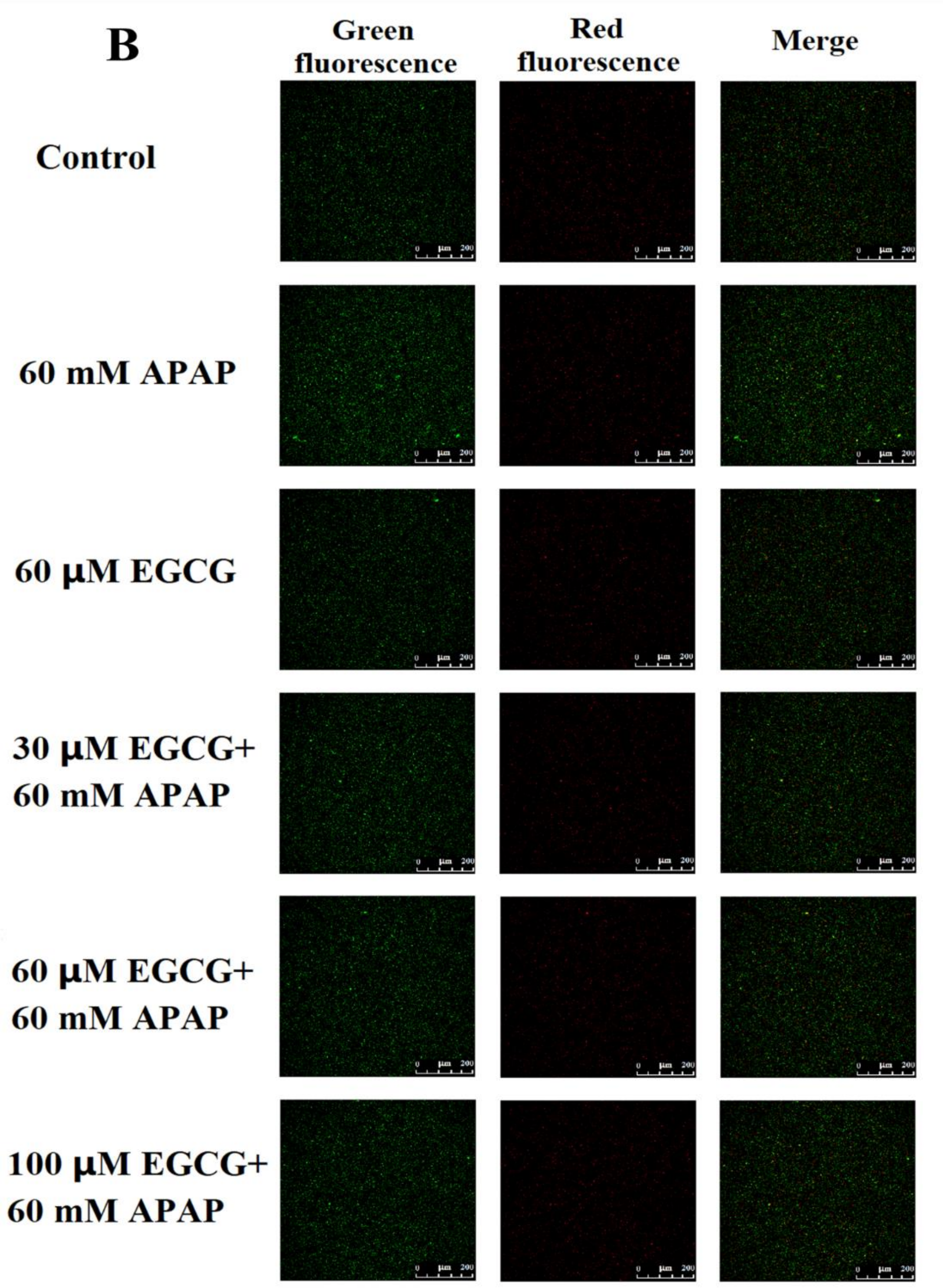

Figure S2. Observe the effects of APAP and EGCG on MMP in rat livers using confocal microscopy.

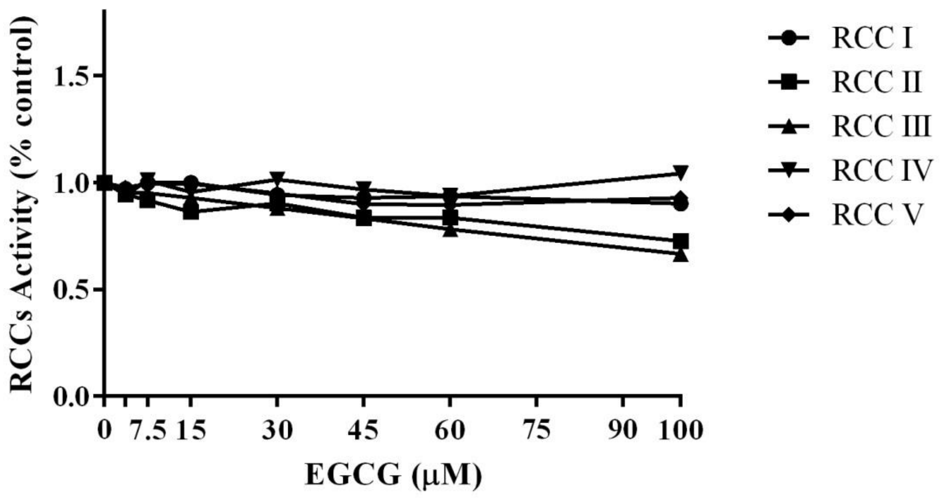

Figure S3. Influence of EGCG on RCC activities in liver mitochondria of SD rats. Values represent the mean ± SD.
